# Soil texture influences soil bacterial biomass in the permafrost-affected alpine desert of the Tibetan plateau

**DOI:** 10.3389/fmicb.2022.1007194

**Published:** 2022-12-12

**Authors:** Ming Shao, Shengyin Zhang, Bin Niu, Yu Pei, Sen Song, Tianzhu Lei, Hanbo Yun

**Affiliations:** ^1^Northwest Institute of Eco-Environment and Resources, Chinese Academy of Sciences, Lanzhou, China; ^2^University of Chinese Academy of Sciences, Beijing, China; ^3^Institute of Tibetan Plateau Research, Chinese Academy of Sciences, Beijing, China; ^4^State Key Laboratory of Frozen Soil Engineering, BeiLu'He Station, Northwest Institute of Eco-Environment and Resources, Chinese Academy of Sciences, Lanzhou, China; ^5^Center for Permafrost (CENPERM), Department of Geosciences and Natural Resource Management, University of Copenhagen, Copenhagen, Denmark; ^6^Department of Earth, Atmospheric and Planetary Sciences, Purdue University, West Lafayette, IN, United States

**Keywords:** soil texture, soil organic carbon, microbial biomass, Tibetan plateau, climate warming, alkaline permafrost regions

## Abstract

Under warm climate conditions, permafrost thawing results in the substantial release of carbon (C) into the atmosphere and potentially triggers strong positive feedback to global warming. Soil microorganisms play an important role in decomposing organic C in permafrost, thus potentially regulating the ecosystem C balance in permafrost-affected regions. Soil microbial community and biomass are mainly affected by soil organic carbon (SOC) content and soil texture. Most studies have focused on acidic permafrost soil (pH < 7), whereas few examined alkaline permafrost-affected soil (pH > 7). In this study, we analyzed soil microbial communities and biomass in the alpine desert and steppe on the Tibetan plateau, where the soil pH values were approximately 8.7 ± 0.2 and 8.5 ± 0.1, respectively. Our results revealed that microbial biomass was significantly associated with mean grain size (MGS) and SOC content in alkaline permafrost-affected soils (*p* < 0.05). In particular, bacterial and fungal biomasses were affected by SOC content in the alpine steppe, whereas bacterial and fungal biomasses were mainly affected by MGS and SOC content, respectively, in the alpine desert. Combined with the results of the structural equation model, those findings suggest that SOC content affects soil texture under high pH-value (pH 8–9) and that soil microbial biomass is indirectly affected. Soils in the alpine steppe and desert are dominated by plagioclase, which provides colonization sites for bacterial communities. This study aimed to highlight the importance of soil texture in managing soil microbial biomass and demonstrate the differential impacts of soil texture on fungal and bacterial communities in alkaline permafrost-affected regions.

## Introduction

Permafrost, defined as soil that has been frozen for >2 consecutive years, covers approximately 24% of the exposed land area in the Northern Hemisphere (Zhang et al., 1999), and stores approximately 44% of the global soil organic carbon (SOC) at a depth of 0–3 m (Schuur et al., 2008; Hugelius et al., 2014). Permafrost-affected regions are exposed to increasing global temperatures, and the resulting permafrost thaw can not only unlock considerable organic carbon (C) but also increase soil microbial activity in these regions (Jansson and Tas, 2014). Moreover, it can accelerate the decomposition of exposed organic C (Schadel et al., 2016). This breakdown of organic C will lead to the release of substantial amounts of CO_2_ and CH_4_ into the atmosphere, potentially contributing to global warming (Liu et al., 2014; Schuur et al., 2015).

As key decomposers of organic C in permafrost (Falkowski et al., 2008; Jansson and Tas, 2014), soil microorganisms can regulate the ecosystem C balance in permafrost-affected regions under warm climate conditions (Graham et al., 2012; Crowther et al., 2019). Therefore, understanding the quantitative information regarding soil communities that drive elemental cycling as well as the environmental conditions that regulate their activity in permafrost-affected regions is important for accurately predicting the permafrost C feedback (Mondav et al., 2014; Monteux et al., 2018; Crowther et al., 2019).

Soil texture, which refers to the relative percentage of sand (fine and coarse sand), silt, and clay particles in soils, has been recognized as a significant factor in controlling the overwhelming majority of soil processes, including structural development, water infiltration, nutrient (e.g., N) retention, and C sequestration and storage (Xia et al., 2020). In particular, soil texture plays a crucial role in the physical and chemical protection of SOC against decomposition by microbes (Nichols, 1984; Kravchenko and Guber, 2017). Considering the microbial resource requirements, it has been suggested that SOC is the dominant factor affecting microbial diversity and biomass (Siciliano et al., 2014; Zhang et al., 2017). However, according to the study by Xia et al. (2020), soil pH is the most important factor for the development of the soil microbial community, followed by soil texture (Xia et al., 2020).

Soil pH affects specific microorganisms, as well as overall microbial biomass, microbial activity, and microbial community structure (Aciego Pietri and Brookes, 2009). Further, it strongly influences the protection of SOC by affecting the point of zero charge of the mineral surface (Mayes et al., 2012; Leinemann et al., 2018). With the increase in soil pH, the soil’s maximum adsorption capacity of organic matter gradually decreases (Mayes et al., 2012). However, most relevant studies have focused on acidic soils, and little attention has been paid to alkaline soils (Chu et al., 2010; Lipson et al., 2015). Therefore, the relative importance of SOC context and soil texture with regard to microbial communities and biomass needs to be further explored for alkaline permafrost-affected soils.

The Tibetan plateau, which is the largest high-altitude permafrost-affected region, contains 15.3 Pg C in the top 3 m of soil (Ding et al., 2016). The permafrost on this plateau is warmer and thinner than that at the high latitudes of North America and Russia, making it more sensitive to climate change (Cheng, 1998; Qin et al., 2021). The distribution areas of the alpine steppe and desert on the Tibetan plateau are 27.54% and 8.96%, ranking first and third, respectively (Yu et al., 2015). Furthermore, the permafrost soil is alkaline (Zhang et al., 2013; Chen et al., 2019), which is different from the acidic soil found in the Arctic region (Mannisto et al., 2007; Chu et al., 2010). Thus, an alluvial fan covered by alpine desert and steppe in the Beilu River Basin on the Tibetan plateau was selected. We hypothesized that SOC content, soil pH, and soil texture affect microbial communities and that SOC is the dominant driving factor for microbial biomass in both ecosystems.

## Materials and methods

### Study area

The study area is located in the permafrost region of the Beilu River Basin on the central Tibetan plateau. The mean annual temperature measured at a meteorological station varies from −5.4°C to −3.6°C (Ni et al., 2021). The area has an arid climate with a mean annual precipitation of 369.8 mm, according to meteorological records, this is much smaller than the mean annual evaporation of 1,317 mm (Peddle and Franklin, 1993). There are three main types of ecosystems: alpine meadow, alpine steppe, and alpine desert (Wu et al., 2015). The alpine meadow site was dominated by *Kobresia pygmaea*, *Kobresia humilis*, *Kobresia capilifolia* and *Polygonum viviparum*. In the alpine steppe area, *Stipa purpurea* and *Carex rigescens* dominated, accounting for approximately 40% of the site (Zhang et al., 2013). The alpine desert surface was not covered by vegetation. The region is under continuous permafrost with an active layer forming in the thawing season, with a maximum depth of 3 m and an average annual ground temperature of −0.9°C (Niu et al., 2005; Yu et al., 2015). Human influence in this area is weak, and the area is largely undisturbed.

### Sampling and preparation of soils

**Figure 1:**
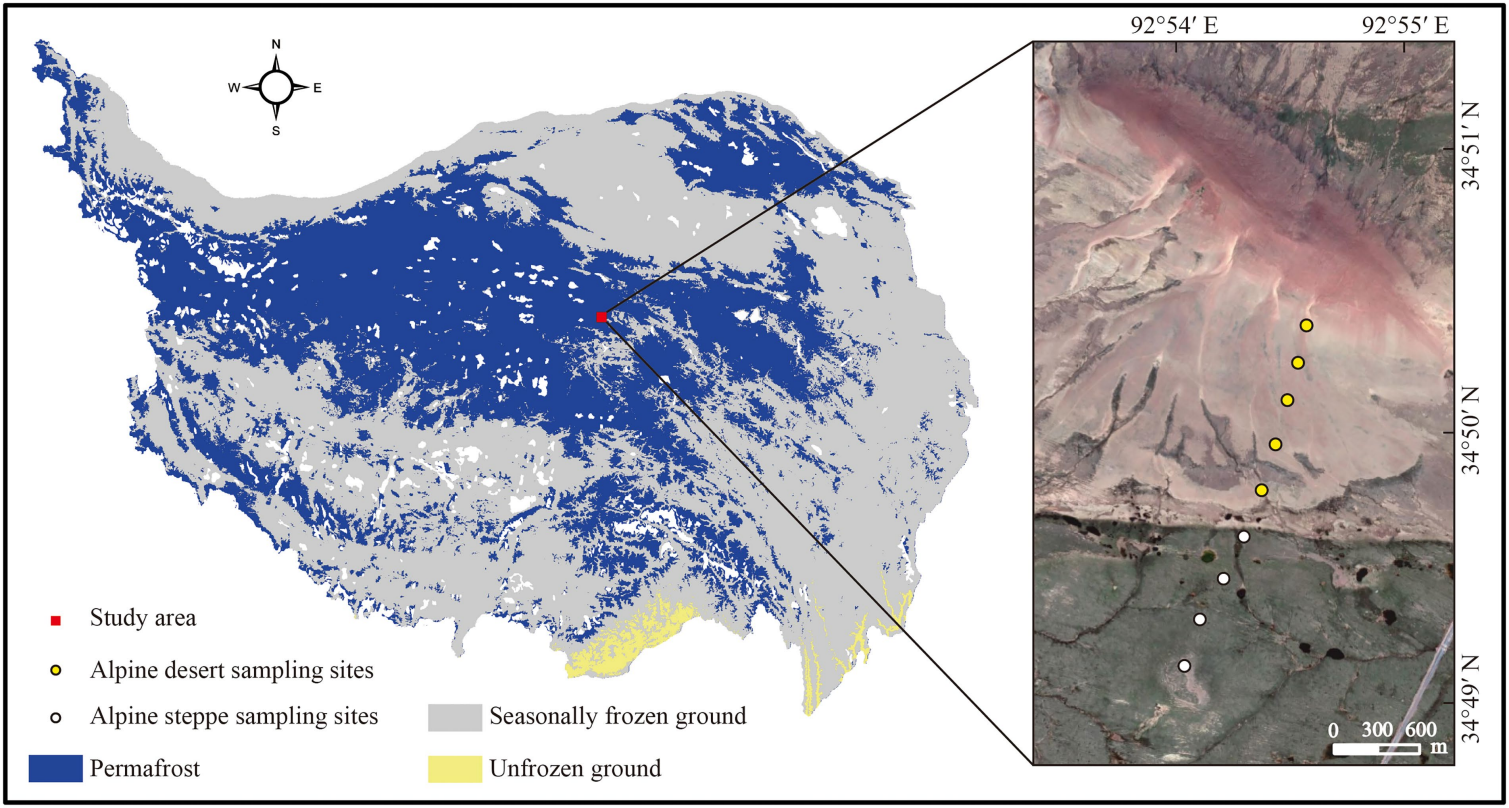
Sampling sites and distribution of permafrost on the Tibetan plateau. The map was created by the authors using ArcMap 10.4 (Environmental Systems Research Institute, Inc., Redlands, CA, USA; Zou et al., 2017; https://tc.copernicus.org/articles/11/2527/2017/).

Twenty-seven soil samples were collected from nine well-separated sites (300–400 m apart) with two types of ecosystems at the Beilu River Long-term Permafrost Research Station (34°50′57.42″N, 92°54′23.96″E, 4694 m) in mid-September 2021. Five sampling sites were selected from the alpine desert, and four were selected from the alpine steppe (Figure 1), and soil from each sampling site was collected in three layers: 0–5 cm, 20–30 cm, and 50–60 cm. At each sampling site, three soil replicates (collected using a soil auger with a diameter of 5 cm) were combined as a single replicating sample. Each collected soil sample was divided into two subsamples after sieving (using a 2 mm mesh) to remove surface vegetation and large particles. The first subsample was stored at −20°C for phospholipid fatty acid (PLFA) analysis, and the remaining part was air-dried to determine the physical and chemical properties of the soil, including pH, SOC content, soil water content (SWC), soil texture, and mineral composition.

### Soil property analyses

Samples were dried for 24 h in an oven at 105°C and then allowed to take up as much moisture, as possible. The SWC of each soil sample was determined using gravimetric analysis (Treat et al., 2014). A pH meter electrode (PB-10, Sartorius, Germany) was used to determine the pH of the soil in a 1:2.5 mixture of soil and deionized water. A DDS-307A conductivity meter (Precision and Scientific Corp. Shanghai, China) was used to measure soil electrical conductivity (EC) in a 1:5 mixture of soil and deionized water. After the samples were acidified with 7% HCl to remove carbonates and rinsed with deionized water until a neutral pH value was obtained, a CS-902G analyzer was used to determine total SOC content. Using a particle size analyzer (Malvern Masterizer 2000, UK) with a measuring range of 0.02–2000 μm and after removing organic matter and carbonates with hydrogen peroxide and HCl, respectively, soil texture (clay/silt/fine sand/coarse sand %) was assessed. Soil particle size was sometimes used to compute the mean grain size (MGS) as follows: MGS = 1/3 (D16 + D50 + D84; Folk and Ward, 1957). By applying Cu − K radiation at 40 kV and 40 mA and operating at a divergence slit and scattering slits of 1° and reception slit of 0.15 mm, mineral composition was determined. The rate was 4°(2θ)/min, step interval was 0.02°, and scanning angle ranged from 2° to 52°. Sample mineral contents and percentages were determined using the MDI Jade 5 program. SY/T 5163–2010, a Chinese industrial standard, was used to determine mineral content (Sun et al., 2020).

### Phospholipid fatty acid extraction and analyses

Using the single-phase Bligh and Dyer technique, PLFAs were isolated from lyophilized soil biomass (Qin et al., 2021). A single-phase combination of chloroform, methanol, and phosphate buffer (1:2:0.8, v/v/v; pH 7.4) was used to extract 5 g of the mixed soil sample twice, and the extracted sample was shaken vigorously each time for at least 2 h. To separate the solution into two phases, water and chloroform were added in equal quantities. On a silica acid column (Unisil, Clarkson Chemical Co., Williamsport, PA, USA), the organic phase was recovered and separated into neutral lipids, glycolipids, and phospholipids *via* sequential elution with chloroform, acetone, and methanol, respectively. Fatty acid methyl esters (FAMEs) were synthesized *via* moderate alkaline methanolysis of the phospholipids, and the PLFA samples were then stored at 20°C until analysis. Each test sample contained an identical quantity of n-tetracosane-D50 (C_24_D_50_), which was added as an internal standard. The isolated PLFAs were subsequently quantified using GC–MS (Agilent 7890A-5,977 N), and the MIDI Sherlock Microbial Identification System was used to identify them (MIDI Inc., Newark, DE, USA). We relied on specific biomarkers to quantify bacteria (i14:0, a15:0, i15:0, i16:0, 16:1ω7c, a17:0, cy17:0, i17:0, 18:1ω7, and cy19:0) and fungi (18,2ω6 and 18:2ω9c; Qin et al., 2021).

### Statistical analyses

Before conducting statistical analyses, the Kolmogorov–Smirnov test and Levene’s test were performed to examine data normality and homogeneity of variance. All factors, except for pH, SWC, EC, MGS, coarse sand, and fine sand, did not conform to a normal distribution and were log-transformed. Variance inflation factor (VIF) was calculated to evaluate the collinearity of the variables (acceptable collinearity was considered as VIF of ≤2; Craney and Surles, 2007; Qin et al., 2019). The above statistical analyses were conducted using SPSS 26.0.0.0, with a significance level of 0.05.

Next, the statistical analyses involved the following five steps. First, we used mixed-effects models (R package: nlme) to examine differences in soil physicochemical properties and PLFA values between the two ecosystems, depth, and interactions between ecosystem and depth (depth × ecosystem). In the model, the fixed factors were ecosystem and soil depth, and the random factor was the sampling site. No interaction was noted between the ecosystem and soil depth. Second, linear regression models were used to determine the relationships between soil physicochemical properties and microbial communities.

Third, to explore the relationships between PLFA biomass and pH, MGS, and SOC content, we used partial correlation analysis, which is considered a successful strategy to evaluate the critical indicators of numerous highly correlated variables (Doetterl et al., 2015). Fourth, the relative importance of different variables for bacterial PLFAs of two types of ecosystems was further analyzed using variation partitioning analysis (VPA; R package: vegan).

Finally, we constructed a structural equation model (SEM; R packages: piecewiseSEM and nlme) to analyze the factors affecting bacterial PLFAs through direct and indirect effects in the alpine desert. Plagioclase content affected soil texture through weathering. The proportion of clay minerals and soil pH had indirect effects on bacterial PLFAs, whereas SOC content showed a direct effect. The overall goodness-of-fit of the model was tested using chi-square test (*χ*^2^) and root mean the square error of approximation (RMSEA). Goodness-of-fit was indicated by a low Chisq value, high probability value (*p* >  0.05), and RMSEA value of approximately 0 (Vieira et al., 2020).

## Results

### Characteristics of soil properties in different ecosystems

Our study identified four soil texture groups: coarse sand (200–2,000 μm), fine sand (20–200 μm), silt (2–20 μm), and clay (< 2 μm). In the alpine desert, coarse sand accounted for 23.2%–32.7%, fine sand accounted for 44.3%–74.2%, silt accounted for 2.3%–15.0%, and clay accounted for 0.1%–11.8%. In the alpine steppe, coarse sand accounted for 1.5%–40.9%, fine sand accounted for 50.0%–87.7%, silt accounted for 2.4%–17.7%, and clay accounted for 0.2%–1.9%. MGS was calculated for the alpine desert and steppe, showing average values of 164.1 and 123.2 μm, respectively (Supplementary Table S1).

**Figure 2:**
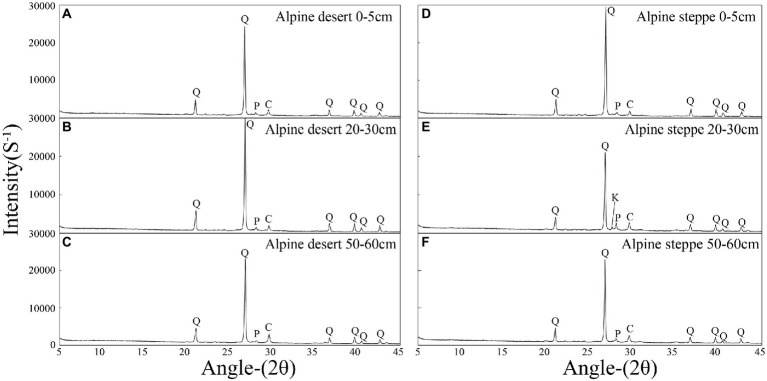
X-ray diffraction patterns of the typical samples. The 0–5 cm layer of the alpine desert **(A)**; the 20–30 cm layer of the alpine desert **(B)**; the 50–60 cm layer of the alpine desert **(C)**; the 0–5 cm layer of alpine steppe **(D)**; the 20–30 cm layer of the alpine steppe **(E)**; and the 50–60 cm layer of the alpine steppe **(F)**. Abbreviations for various minerals: Q-, quartz; C-, calcite; P-, plagioclase; K-, potassium feldspar.

Mineralogy of the soils was analyzed and all samples showed a similar mineralogical composition (Figure 2). Quartz was the dominant mineral in the alpine desert (70.7%–89.2%), and other minerals included calcite (4.4%–20.4%), plagioclase (2.4%–5.6%), K-feldspar (0.0%–5.5%), and clay minerals (0.8%–8.3%). Further, quartz was the dominant mineral in the alpine steppe (55.5%–88.5%), and other minerals included calcite (3.5%–17.3%), plagioclase (3.9%–15.2%), K-feldspar (0.0%–10.5%), and clay minerals (0.0%–12.6%; Supplementary Table S1). The clay mineral contents of 22 samples were below the detection limit of the instrument, which was consistent with the results of soil particle size analysis (Supplementary Table S1).

All 27 soil samples were alkaline in nature, with pH values of 8.3–9.0 and 8.3–8.8 in the alpine desert and steppe, respectively. The SWC of the alpine desert ranged from 3.5% to 23.4%, whereas that of the alpine steppe ranged from 6.7% to 29.4%. SOC content of the alpine desert ranged from 0.4‰ to 8.3‰, and that in the alpine steppe ranged from 1.4‰ to 6.8‰ (Supplementary Table S1).

There were fundamental differences in the physicochemical properties of the soil from the two ecosystems. According to the mixed-effects model, the above soil parameters did not exhibit significant differences among the three layers, whereas most soil parameters varied significantly between the ecosystems (Supplementary Table S2). Soil pH, quartz content, MGS, and coarse sand content in the alpine desert were significantly higher than those in the alpine steppe (all *p* < 0.05), whereas SWC, EC, SOC content, plagioclase content, and fine sand content showed the opposite results (all *p* < 0.05). There were no significant differences in the contents of K-feldspar, calcite, clay minerals, silt particles, and clay particles between the two ecosystems (all *p* > 0.05). In addition, except for plagioclase content (*p* < 0.05), other soil parameters were not significantly different in terms of depth × ecosystem interaction (all *p* > 0.05). Pearson correlation analysis revealed strong correlations among different environmental variables in the alpine desert compared with those in the alpine steppe (Supplementary Table S3). Elevation was negatively correlated with SWC and EC and positively correlated with pH and coarse sand content (all *p* < 0.05), in the alpine steppe, elevation was only significantly correlated with SOC content (Supplementary Table S3).

### Variation in microbial biomasses in different ecosystems

**Figure 3:**
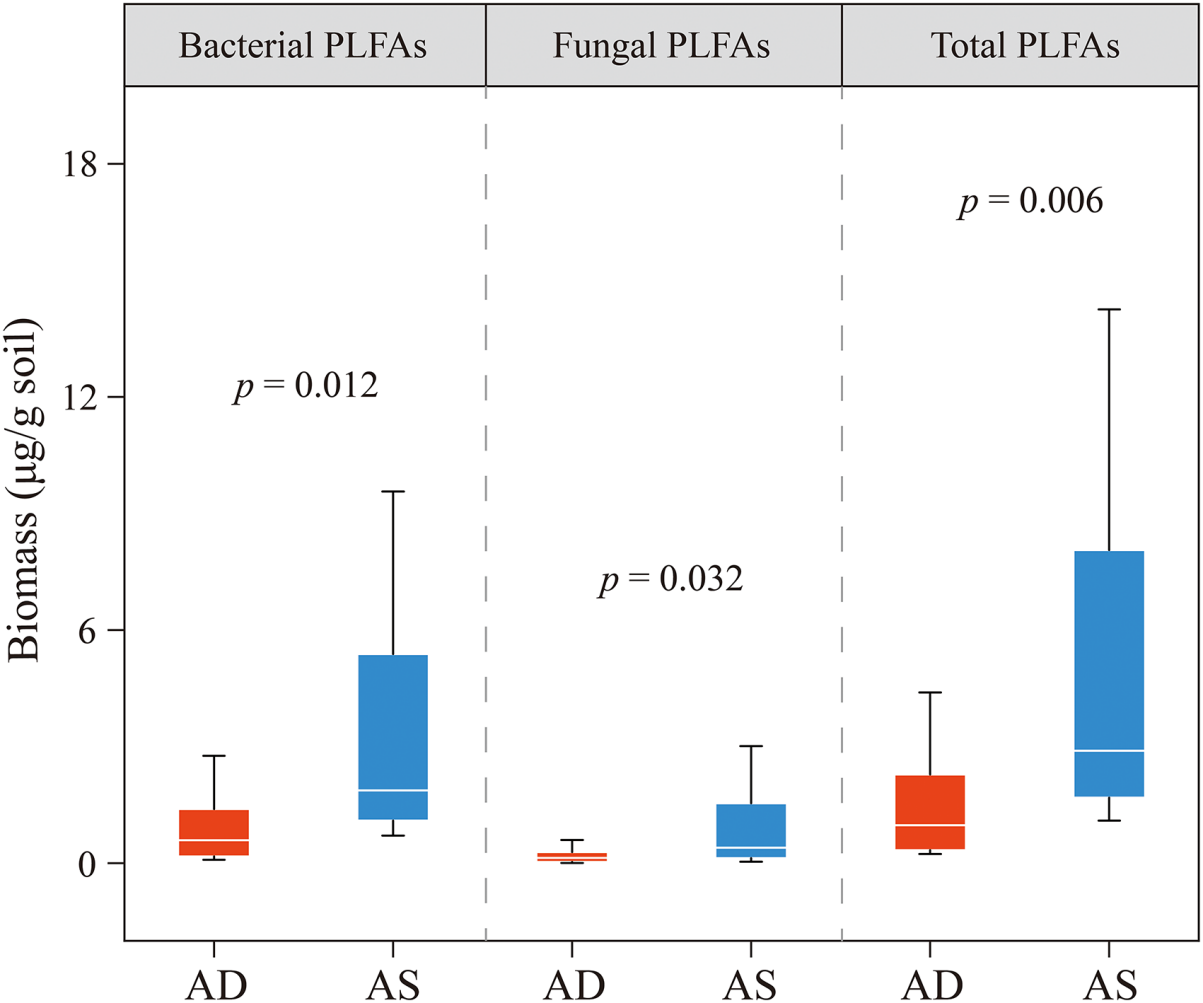
Phospholipid fatty acid (PLFA) contents of bacteria, fungi, and total microorganisms in the alpine desert (AD) and alpine steppe (AS). The ends of the boxes in the box plot represent the 25^th^ and 75^th^ percentiles. Horizontal lines and whiskers within each box indicate the median and standard deviation, respectively (*n* [AD] = 15, *n* [AS] = 12). *p* < 0.05 indicates a significant difference in PLFA content between the alpine desert and steppe.

The average content of total PLFA in the alpine steppe was estimated as 5.2 (μg/g dry soil), which was significantly higher than that in the alpine desert (1.6 μg/g soil; *p* < 0.05; Figure 3). No significant interactions were observed between the ecosystem and depth, suggesting that the effect of the ecosystem on total PLFA content was independent of the sampling depth (*p* = 0.41; Table 1). To further explore the PLFA characteristics of various microbial communities, bacterial and fungal PLFA contents were analyzed. Similarly, the bacterial and fungal PLFA contents in the alpine steppe were significantly higher than those in the alpine desert (*p* < 0.05; Figure 3) without any interaction effect (*p* = 0.40 and 0.37, respectively; Table 1). In addition, the biomass of bacteria was higher than that of fungi in different ecosystems (Figure 3).

**Table 1:** Variance analysis of phospholipid fatty acid (PLFA) contents for different ecosystems and depths.

			Bacterial PLFAs (μg/g soil)		Fungal PLFAs (μg/g soil)		Total PLFAs (μg/g soil)	
	NumDF	DenDF	*F*	*P*	*F*	*P*	*F*	*P*
Intercept	1	24	31.277	<0.001	17.607	<0.001	32.984	<0.001
Depth	2	23	2.375	0.115	2.302	0.123	2.299	0.123
**Ecosystem**	1	24	7.164	**0.012**	5.104	**0.031**	7.734	**0.006**
Depth × ecosystem	5	20	0.991	0.389	1.039	0.372	0.923	0.413

NumDF: numerator degrees of freedom, DenDF: denominator degrees of freedom. The bold values indicate significant differences (*p* < 0.05).

### Drivers of microbial biomasses in different ecosystems

**Figure 4:**
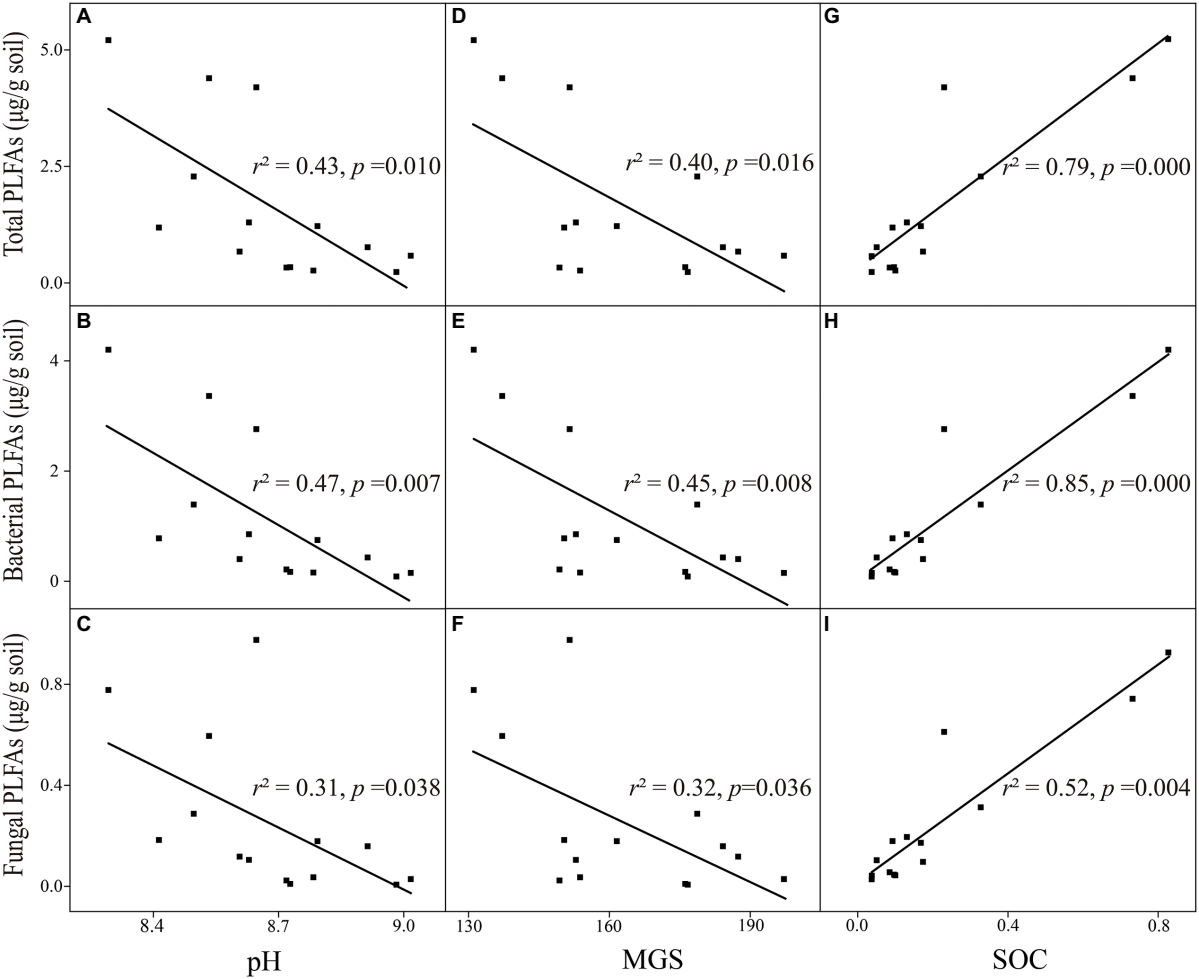
Relationships between environmental factors and microbial properties in the alpine desert. (**A–C**) pH with total PLFAs, bacterial PLFAs and fungal PLFAs. (**D–F**) mean grain size (MGS) with total PLFAs, bacterial PLFAs and fungal PLFAs. (**G–I**) soil organic carbon (SOC) with total PLFAs, bacterial PLFAs and fungal PLFAs. R squared (r^2^) represents the fit degree of linear regression, *p* < 0.05 indicates significant correlation.

We established relationships between microbial properties and soil physicochemical factors. Total, bacterial, and fungal PLFA contents were significantly associated with pH, MGS, and SOC content in the alpine desert (Figure 4). In particular, microbial PLFA content in the alpine desert was negatively correlated with soil texture characterized by MGS (all *p* < 0.05; Figures 4D−F). Higher microbial biomass was observed when the soil pH values were closer to the neutral pH value (all pH > 7; all *p* < 0.05; Figures 4A−C), and microbial biomass increased with the SOC content (all *p* < 0.01; Figures 4G−I).

**Figure 5:**
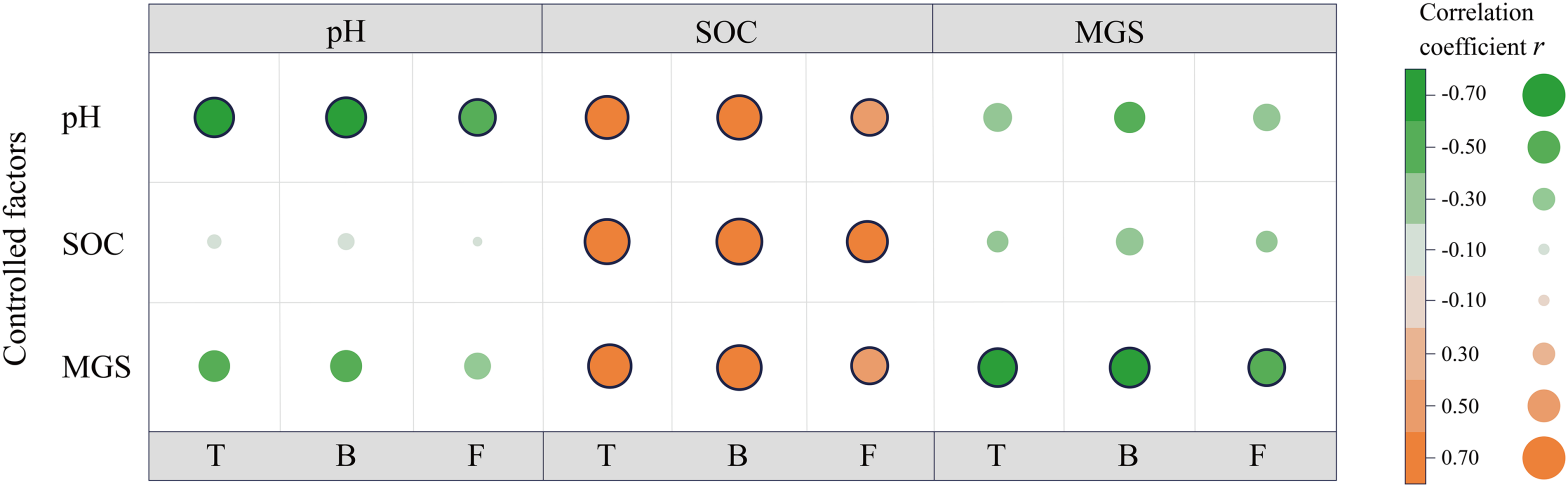
Partial correlations between microbial properties and environmental factors in the alpine desert. Pearson correlation coefficients between the environmental factors (pH, soil organic carbon [SOC] and mean grain size [MGS]) and microbial properties (total phospholipid fatty acids [PLFAs; T], bacterial PLFAs [B], fungal PLFAs [F]) are displayed at the intersection of the same environmental factors in the matrix. The colors of the points indicate the strength and sign of the correlation, and the black frame line indicates the significant correlation at the significance level of 0.05. The color differences observed between the correlations (matrix) and controlled factors indicate the extent to which the correlation between microbial properties and the examined factors is influenced by the control variables (no change in point color between the partial correlations and correlations = no dependency; a decrease/increase in point color intensity = loss/gain of correlation).

Next, the partial correlation analysis between microbial properties and pH, SOC content, and MGS was performed for the alpine desert (Figure 5). Despite the significant correlations of microbial properties with these three factors (Figure 5), the correlation coefficients of bacterial PLFA content with pH and MGS decreased by 78.0% and 51.9%, respectively, after controlling for the role of SOC. In contrast, SOC content was always significantly associated with bacterial PLFA content (all *p* < 0.01) even after controlling for the other two factors. Moreover, after controlling for the role of soil pH or MGS, the correlation coefficients between bacterial PLFA content and another factor decreased significantly. Consistently, total and fungal PLFA contents showed patterns similar to that of bacterial PLFA content in the partial correlation analysis. This demonstrated that SOC content had a direct positive effect on microbial properties, whereas soil pH and MGS showed an indirect effect by negatively affecting microbial properties. In the alpine steppe, microbial biomasses were significantly positively correlated with SOC content (Table 2). There were no significant correlations between other soil environmental factors and microbial biomass, except for a significant positive correlation between fungal biomass and EC. This demonstrates that SOC is the dominant factor affecting microbial biomass in the alpine steppe.

**Table 2:** Pearson correlation coefficients between soil physicochemical properties and microbial biomasses in the alpine steppe.

	pH	SWC	EC	SOC	Quartz	K-Feldspar	Plagioclase	Calcite	Clay minerals	MGS	Coarse sand	Fine sand	Silt	Clay
Total PLFAs (μg/g soil)	−0.560	−0.011	0.544	0.773**	0.111	−0.296	0.383	−0.209	−0.346	−0.210	−0.345	0.402	−0.169	−0.238
Bacterial PLFAs (μg/g soil)	−0.546	−0.021	0.542	0.778**	0.116	−0.285	0.357	−0.205	−0.349	−0.208	−0.331	0.377	−0.145	−0.207
Fungal PLFAs (μg/g soil)	−0.528	0.090	0.577*	0.727**	0.047	−0.319	0.466	−0.159	−0.330	−0.230	−0.376	0.439	−0.184	−0.271

Asterisks indicate significant influence (**indicates *p* < 0.01; *indicates *p* < 0.05).

The bold values indicate significant differences (*p* < 0.05).

### Dominant factors influencing microbial biomasses in different ecosystems

**Figure 6:**
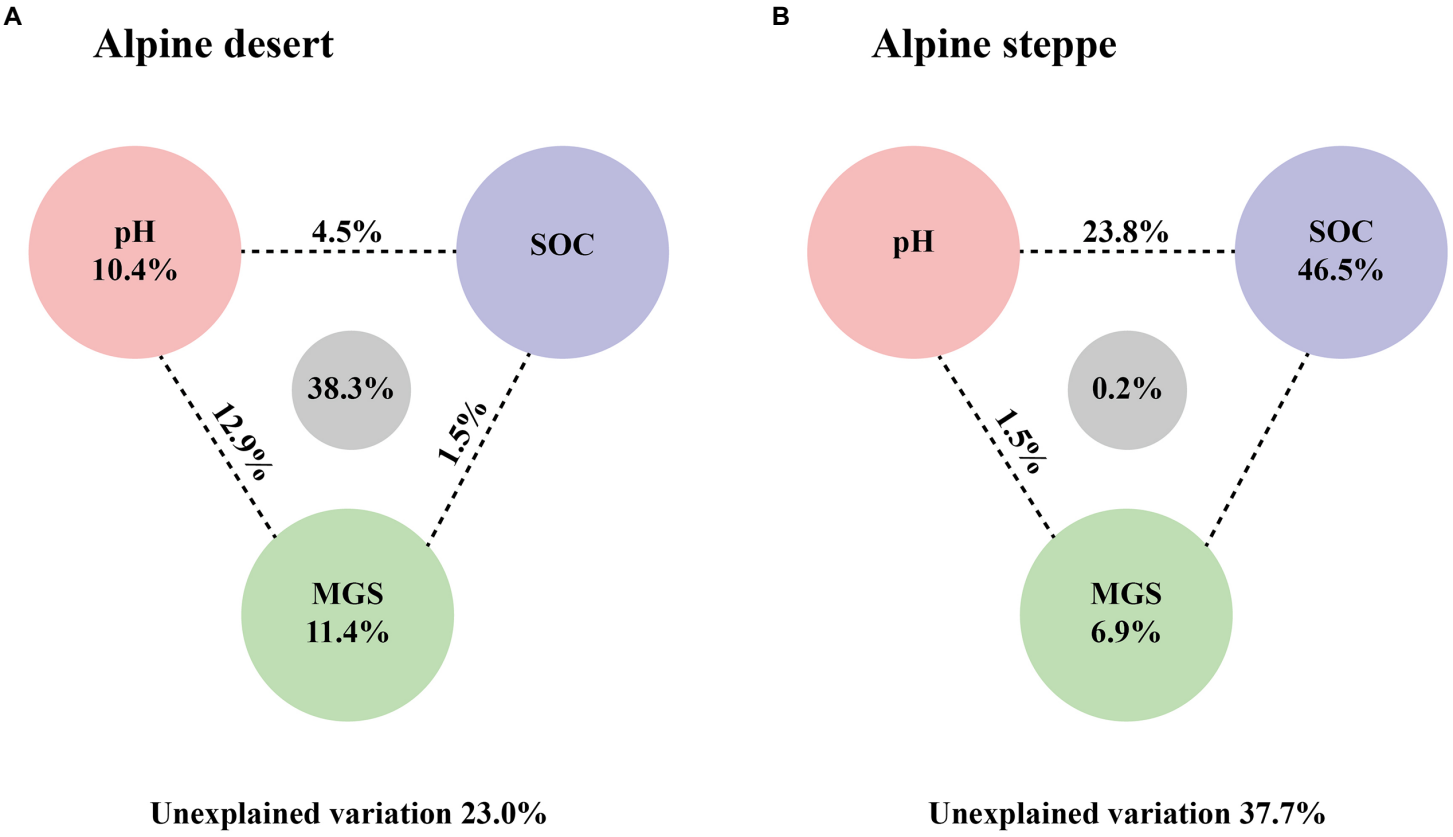
Variation partitioning analysis (VPA) for bacterial PLFAs in the alpine desert **(A)** and in the alpine steppe **(B)**. The numbers between the circles indicate the intersections between the two types of variables on either side.

The dominant factor affecting bacterial biomass was different in the two ecosystems. To further explore the relative importance of soil pH, SOC content, and MGS, VPA was performed to determine their relative effects on microbial biomass. Soil pH, SOC content, and MGS accounted for 67.0% of total effects in the alpine desert, with 10.4% and 11.4% of unique effects presented by pH and MGS, respectively. The results showed that soil MGS had an important influence on bacterial biomass (Figure 6A). In the alpine steppe, bacterial biomass was only significantly affected by SOC content (*R*^2^ = 0.61, *p* < 0.01, Supplementary Table S2). Moreover, VPA revealed that SOC content was the dominant factor affecting bacterial biomass (Figure 6B). Furthermore, SOC content was the dominant factor affecting total microbial and fungal biomasses in both ecosystems (Figure 4; Supplementary Figures S1, S2).

### Soil texture effects on bacterial biomass

**Figure 7:**
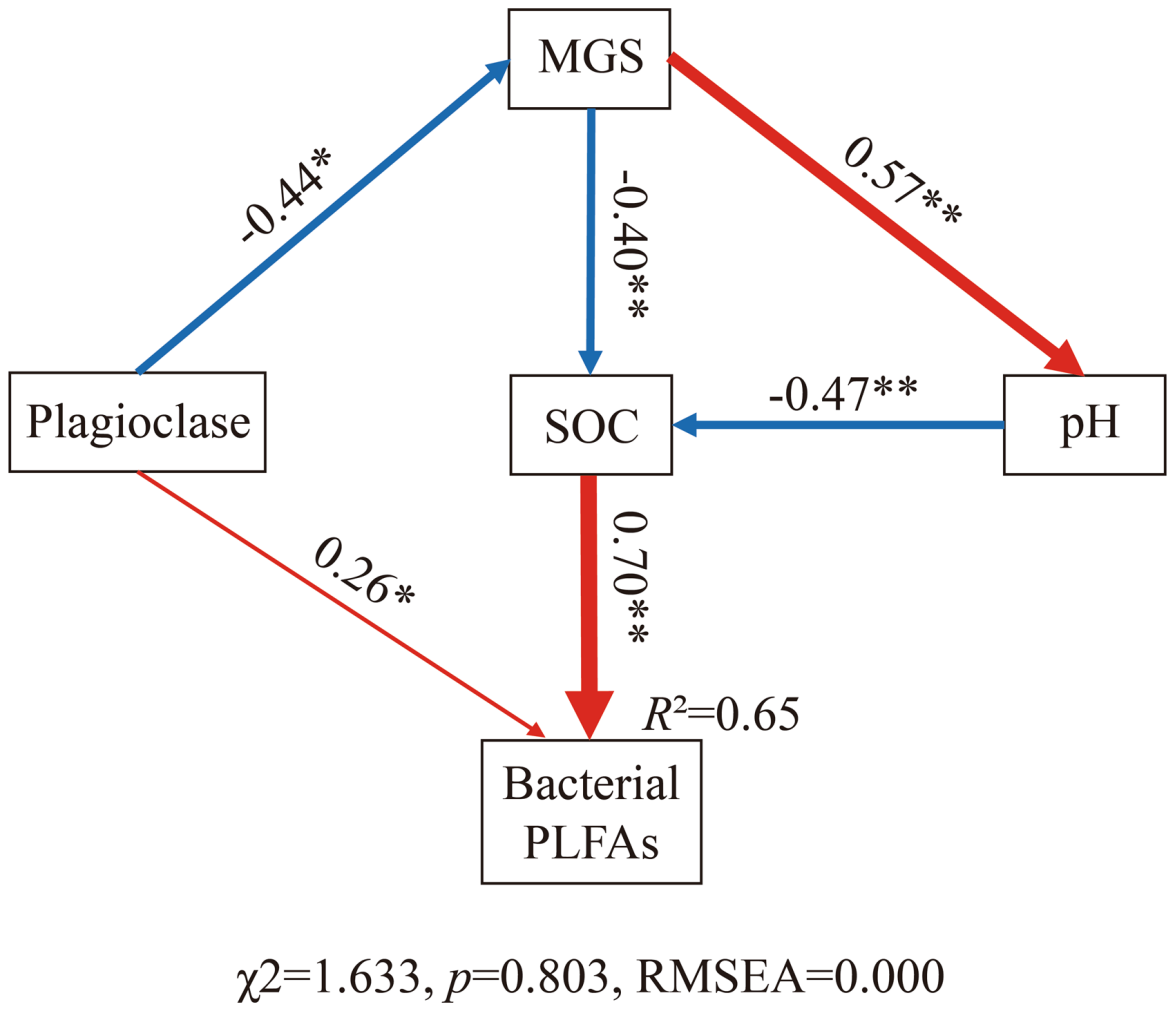
Structural equation models (SEM) showing the multivariate effects on soil bacterial PLFA content. Red solid arrows represent positive significant relationships, and blue solid arrows indicate negative significant relationships. The widths of the red and blue arrows are proportional to the strength of the relationship. The numbers adjacent to the arrows are the standardized path coefficients. *p < 0.05, **p < 0.01.

We assessed the potential drivers of bacterial biomass in further detail by constructing SEMs for all samples of the two ecosystems. The model with the best goodness-of-fit showed that a change in SOC content was the strongest direct driver among all variables affecting bacterial biomass (Figure 6). Owing to their effect on SOC content, soil MGS and pH have additional indirect effects on bacterial PLFA content. Notably, the content of plagioclase in the soil not only had a direct effect on bacterial biomass but also had indirect effects *via* MGS. In contrast, no direct link between soil pH and bacterial biomass could be identified (Figure 7).

The nutritive properties of SOC determined its importance in controlling the number of microbial players (Siciliano et al., 2014). Soil pH affected bacterial communities in different ways. Soil pH had a strong direct effect on bacterial community structure and diversity (Fierer and Jackson, 2006; Lauber et al., 2009; Chong et al., 2010; Zinger et al., 2011) and had an indirect effect on the bacterial biomass through organic matter content. The unstable light fraction of organic C content increased at an exponential rate as soil pH changed from alkaline to neutral (Wang et al., 2006; Zhang et al., 2014). This may explain why soil pH was significantly negatively correlated with bacterial PLFA content in the Pearson correlation analysis but no direct effect was observed between the two in the SEM results. Soil texture can indirectly affect bacteria biomass by affecting pH because soil texture is formed through the physical weathering of soil particles (e.g., plagioclase), which results in the chemical weathering of smaller particles and the release of ions that alter the soil pH (Siciliano et al., 2014). Interestingly, the plagioclase content not only controlled soil texture but also affected bacterial biomass. Previous studies have reported that the presence of limiting nutrients in minerals in nutrient-limited environments can allow preferential colonization by differently structured microbial communities (Carson et al., 2009). In soils with limited phosphorus and iron content, diverse microbial communities preferentially colonize silicate minerals that contain these nutrients compared with minerals without these elements (Roberts, 2004; Mauck and Roberts, 2007). This suggests that plagioclase provides colonization sites for bacterial communities in soils.

## Discussion

### Soil texture impacts microbial biomass in the alpine desert

Our results revealed that soil pH and MGS were significantly negatively correlated with total microbial, bacterial, and fungal biomasses. With the increase in soil pH and particle size, the bacteria and fungi biomasses significantly decreased. These changes in microbial biomass may be associated with the double protection of SOC induced by soil texture. Finer textured soils are inherently more aggregated and have finer pore sizes, resulting in increasing physical retention of organic matter in micro aggregates (50–250 μm); this also hinders the entry of microbial enzymes into soils, thus an increase in retention time of organic matter (Hassink, 1992; Six et al., 2002). However, as clay content increases, most of the organic matter binds to fine mineral particles, slowing down the rate of organic matter decomposition (Krull et al., 2003). In addition, our results showed a significant negative correlation between MGS and SOC in the alpine desert (Table 2). The soil texture controlled the availability of organic matter in the soil, which indirectly affected the soil microbial biomass. There was no significant correlation between MGS and microbial biomass in the alpine steppe (Supplementary Table S2). This may be because of the destabilizing effect of plant C input on C stocks in the alpine steppe through priming effects (Fontaine et al., 2007; Shahzad et al., 2018; Chen et al., 2019). Plant C input accelerated the turnover of organic C by promoting microbial growth and nitrogen demand and disrupting the protective mineral–organic associations, resulting in the destabilization of soil organic matter (Craine et al., 2007; Keiluweit et al., 2015; Jiang et al., 2021).

### Differences in dominant factors influencing microbial biomass in the alpine desert

The correlation between soil physicochemical factors and microbial biomass was supported by the significant change in microbial biomass when compared to soil pH, MGS, and SOC content in the alpine desert (Table 2; Figure 4). VPA analysis revealed that SOC was the dominant driver of bacterial biomass in the alpine steppe and of fungal biomass in both ecosystems (Figure 6B; Supplementary Figure S2). SOC played a pivotal role in soil fertility, potentially providing the nutritional properties that make a community more suitable for adaptive microbial taxa to grow, dominate, and repel other members of the community (Hardin, 1960; Siciliano et al., 2014). As an important intermediate between plants and microorganisms, SOC is also the dominant factor affecting microbial diversity and activity (Benizri and Amiaud, 2005; Eilers et al., 2010). Several studies have confirmed that the SOC content can directly influence the dynamics of microbial community structure and growth (Liu et al., 2014; Zhang et al., 2014). Finally, SOC could also indirectly influence microbial communities through its effect on plant diversity as microorganisms depend upon plant litter and root exudates (Millard and Singh, 2010).

In contrast with our hypothesis, soil MGS, but not SOC, was the primary driver affecting bacterial biomass in the alpine desert. Soil texture exerted tandem effects on microbial communities by affecting resources, moisture, and pore size (Bronick and Lal, 2005). Our results showed that the soil texture in the alpine desert was coarser than that in the alpine steppe (Table 1). Coarse soils hold less water than finer soils owing to a greater proportion of macropores in coarse soils, resulting in lower rates of resource diffusion and bacterial migration through pores (Mtambanengwe et al., 2004). However, fungi can acquire and redistribute nutrients from great distances through hyphal elongation and branching in macropores (Otten et al., 2001; Cairney, 2005; Guhr et al., 2015). Therefore, macropores confined bacteria more than fungi due to being exposed to drought and more frequent resource loss. In addition, bacteria, alleged r-strategy organisms, prefer nutrient-rich environments and may therefore be sensitive to nutrient changes in nutrient-restricted environments (Xia et al., 2020). These factors might explain why in the resource-poor alpine desert, MGS was the dominant factor affecting bacterial PLFAs, and SOC was the dominant factor affecting fungal PLFAs.

## Conclusion

This study showed that there were significant differences in the physicochemical and microbial properties of soils from the two ecosystems (*p* < 0.01). Plant C input could mask the effects of soil texture on microbes. SOC was the dominant factor affecting bacterial biomass in the alpine steppe and fungal biomass in both ecosystems, whereas soil texture had a significant influence on bacterial biomass in the alpine desert. Our results provide evidence that finer soil particles are more conducive to the development of soil microorganisms. It was also speculated that plagioclase provides colonization sites for bacterial communities. Therefore, further model development should incorporate the effects of soil texture on SOC content and soil microorganisms to accurately predict permafrost C dynamics and its associated climate feedback.

Although our study demonstrated the influence of soil texture on soil bacterial biomass in the alpine steppe, there are still some limitations that need to be addressed in future studies. In particular, despite being a frequently used approach in microbial experiments, the PLFA extraction method could not accurately determine the functional expression of microorganisms. Metagenomic sequencing should be used to further evaluate the effect of soil texture on soil microorganisms. In addition, we selected an alluvial fan associated with single provenance to examine the influence of soil texture on microbial biomass in this study, without considering the influence of different mineral compositions on microbial communities under different bedrock backgrounds. More empirical studies with diverse provenances and bedrock backgrounds are warranted to further advance our understanding of this issue.

## Data availability statement

The original contributions presented in the study are included in the article/Supplementary material, further inquiries can be directed to the corresponding author.

## Author contributions

MS: conceptualization, methodology, software, writing—original draft, data curation, investigation, and experiment. SZ: writing—review and editing, methodology, investigation, and data curation. BN: investigation, and writing—review and editing. YP: data curation, and methodology. SS: investigation. TL: resources—instrumentation, funding acquisition. HY: conceptualization, and writing—review and editing. All authors contributed to the article and approved the submitted version.

## Funding

This work was supported by the National Natural Science Foundation of China (grant numbers 41072107, and 42002174) and the Natural Science Foundation of Gansu Province, China (grant number 20JR10RA030).

## Conflict of interest

The authors affirm that they have no known financial or interpersonal conflicts that would impact the research presented in this study.

## Publisher’s note

All claims expressed in this article are solely those of the authors and do not necessarily represent those of their affiliated organizations, or those of the publisher, the editors and the reviewers. Any product that may be evaluated in this article, or claim that may be made by its manufacturer, is not guaranteed or endorsed by the publisher.

## References

[ref1] Aciego Pietri J. C. Brookes P. C. (2009). Substrate inputs and pH as factors controlling microbial biomass, activity and community structure in an arable soil. Soil Biol. Biochem. 41, 1396–1405. doi: 10.1016/j.soilbio.2009.03.017

[ref2] Benizri E. Amiaud B. (2005). Relationship between plants and soil microbial communities in fertilized grasslands. Soil Biol. Biochem. 37, 2055–2064. doi: 10.1016/j.soilbio.2005.03.008, PMID: 22962602

[ref3] Bronick C. J. Lal R. (2005). Soil structure and management: a review. Geoderma 124, 3–22. doi: 10.1016/j.geoderma.2004.03.005, PMID: 36384845

[ref4] Cairney J. W. G. (2005). Basidiomycete mycelia in forest soils: dimensions, dynamics and roles in nutrient distribution. Mycol. Res. 109, 7–20. doi: 10.1017/S0953756204001753, PMID: 15736859

[ref5] Carson J. K. Campbell L. Rooney D. Clipson N. Gleeson D. B. (2009). Minerals in soil select distinct bacterial communities in their microhabitats. FEMS Microbiol. Ecol. 67, 381–388. doi: 10.1111/j.1574-6941.2008.00645.x, PMID: 19187213

[ref6] Chen L. Y. Liu L. Qin S. Q. Yang G. B. Fang K. Zhu B.. (2019). Regulation of priming effect by soil organic matter stability over a broad geographic scale. Nat. Commun. 10:10. doi: 10.1038/s41467-019-13119-z 31704929 PMC6841703

[ref001] Cheng G. D. (1998). Glaciology and Geocryology of China in the Past 40 Years: progress and Prospect. J Glaciol Geocryol. 20, 213–226.

[ref7] Chong C. W. Pearce D. A. Convey P. Tan G. Y. A. Wong R. C. S. Tan I. K. P. (2010). High levels of spatial heterogeneity in the biodiversity of soil prokaryotes on Signy Island, Antarctica. Soil Biol. Biochem. 42, 601–610. doi: 10.1016/j.soilbio.2009.12.009

[ref02] Chu H. Fierer N. Lauber C. L. Caporaso J. G. Knight R. Grogan P. (2010). Soil bacterial diversity in the Arctic is not fundamentally different from that found in other biomes. Environmental Microbiology 12, 2998–3006. doi: 10.1111/j.1462-2920.2010.02277.x 20561020

[ref8] Craine J. M. Morrow C. Fierer N. (2007). Microbial nitrogen limitation increases decomposition. Ecology 88, 2105–2113. doi: 10.1890/06-1847.1, PMID: 17824441

[ref9] Craney T. A. Surles J. G. (2007). Model-dependent variance inflation factor Cutoff values. Qual. Eng. 14, 391–403. doi: 10.1081/QEN-120001878

[ref03] Crowther T. W. van den Hoogen J. Wan J. Mayes M. A. Keiser A. D. Mo L.. (2019). The global soil community and its influence on biogeochemistry. Science 365:aav0550. doi: 10.1126/science.aav0550 31439761

[ref04] Ding J. Z. Li F. Yang G. B. Chen L. Y. Zhang B. B. Liu L.. (2016). The permafrost carbon inventory on the Tibetan Plateau: a new evaluation using deep sediment cores. Global Change Biology 22, 2688–2701. doi: 10.1111/gcb.13257 26913840

[ref10] Doetterl S. Stevens A. Six J. Merckx R. Van Oost K. Pinto M. C.. (2015). Soil carbon storage controlled by interactions between geochemistry and climate. Nat. Geosci. 8:780–783. doi: 10.1038/ngeo2516

[ref11] Eilers K. G. Lauber C. L. Knight R. Fierer N. (2010). Shifts in bacterial community structure associated with inputs of low molecular weight carbon compounds to soil. Soil Biol. Biochem. 42, 896–903. doi: 10.1016/j.soilbio.2010.02.003

[ref01] Falkowski P. G. Fenchel T. Delong E. F. (2008). The microbial engines that drive Earth’s biogeochemical cycles. Science 320, 1034–1039. doi: 10.1126/science.1153213 18497287

[ref12] Fierer N. Jackson R. B. (2006). The diversity and biogeography of soil bacterial communities. Proc. Natl. Acad. Sci. U. S. A. 103, 626–631. doi: 10.1073/pnas.0507535103, PMID: 16407148 PMC1334650

[ref13] Folk R. L. Ward W. C. (1957). Brazos River Bar: a study in the significance of grain size parameters. J. Sediment. Res. 27, 3–26. doi: 10.1306/74D70646-2B21-11D7-8648000102C1865D

[ref14] Fontaine S. Barot S. Barre P. Bdioui N. Mary B. Rumpel C. (2007). Stability of organic carbon in deep soil layers controlled by fresh carbon supply. Nature 450, 277–U10. doi: 10.1038/nature06275, PMID: 17994095

[ref05] Graham D. E. Wallenstein M. D. Vishnivetskaya T. A. Waldrop M. P. Phelps T. J. Pfiffner S. M.. (2012). Microbes in thawing permafrost: the unknown variable in the climate change equation. Isme Journal 6, 709–712. doi: 10.1038/ismej.2011.163 22094350 PMC3309365

[ref15] Guhr A. Borken W. Spohn M. Matzner E. (2015). Redistribution of soil water by a saprotrophic fungus enhances carbon mineralization. Proc. Natl. Acad. Sci. U. S. A. 112, 14647–14651. doi: 10.1073/pnas.1514435112, PMID: 26554004 PMC4664368

[ref16] Hardin G. (1960). Competitive exclusion principle. Science 131, 1292–1297. doi: 10.1126/science.131.3409.1292, PMID: 14399717

[ref17] Hassink J. (1992). Effects of soil texture and structure on carbon and nitrogen mineralization in grassland soils. Biol. Fertil. Soils 14, 126–134. doi: 10.1007/BF00336262

[ref06] Hugelius G. Strauss J. Zubrzycki S. Harden J. W. Schuur E. A. G. Ping C.-L.. (2014). Estimated stocks of circumpolar permafrost carbon with quantified uncertainty ranges and identified data gaps. Biogeosciences 11, 6573–6593. doi: 10.5194/bg-11-6573-2014

[ref18] Jansson J. K. Tas N. (2014). The microbial ecology of permafrost. Nat. Rev. Microbiol. 12, 414–425. doi: 10.1038/nrmicro3262, PMID: 24814065

[ref19] Jiang Z. H. Liu Y. Z. Yang J. P. Brookes P. C. Gunina A. (2021). Rhizosphere priming regulates soil organic carbon and nitrogen mineralization: the significance of abiotic mechanisms. Geoderma 385:9. doi: 10.1016/j.geoderma.2020.114877

[ref20] Keiluweit M. Bougoure J. J. Nico P. S. Pett-Ridge J. Weber P. K. Kleber M. (2015). Mineral protection of soil carbon counteracted by root exudates. Nat. Clim. Chang. 5, 588–595. doi: 10.1038/nclimate2580

[ref21] Kravchenko A. N. Guber A. K. (2017). Soil pores and their contributions to soil carbon processes. Geoderma 287, 31–39. doi: 10.1016/j.geoderma.2016.06.027

[ref22] Krull E. S. Baldock J. A. Skjemstad J. O. (2003). Importance of mechanisms and processes of the stabilisation of soil organic matter for modelling carbon turnover. Funct. Plant Biol. 30, 207–222. doi: 10.1071/FP02085, PMID: 32689005

[ref23] Lauber C. L. Hamady M. Knight R. Fierer N. (2009). Pyrosequencing-based assessment of soil pH as a predictor of soil bacterial community structure at the continental scale. Appl. Environ. Microbiol. 75, 5111–5120. doi: 10.1128/AEM.00335-09, PMID: 19502440 PMC2725504

[ref07] Leinemann T. Preusser S. Mikutta R. Kalbitz K. Cerli C. Hoschen C.. (2018). Multiple exchange processes on mineral surfaces control the transport of dissolved organic matter through soil profiles. Soil Biology and Biochemistry 118, 79–90. doi: 10.1016/j.soilbio.2017.12.006

[ref08] Lipson D. A. Raab T. K. Parker M. Kelley S. T. Brislawn C. J. Jansson J. (2015). Changes in microbial communities along redox gradients in polygonized Arctic wet tundra soils. Environ. Microbiol. Rep. 7, 649–657. doi: 10.1111/1758-2229.12301 26034016

[ref24] Liu J. J. Sui Y. Y. Yu Z. H. Shi Y. Chu H. Y. Jin J.. (2014). High throughput sequencing analysis of biogeographical distribution of bacterial communities in the black soils of Northeast China. Soil Biol. Biochem. 70, 113–122. doi: 10.1016/j.soilbio.2013.12.014

[ref09] Mannisto M. K. Tiirola M. Haggblom M. M. (2007). Bacterial communities in Arctic fjelds of Finnish Lapland are stable but highly pH-dependent. Fems Microbiology Ecology 59, 452–465. doi: 10.1111/j.1574-6941.2006.00232.x 17328122

[ref25] Mauck B. S. Roberts J. A. (2007). Mineralogic control on abundance and diversity of surface-adherent microbial communities. Geomicrobiol J. 24, 167–177. doi: 10.1080/01490450701457162

[ref010] Mayes M. A. Heal K. R. Brandt C. C. Phillips J. R. Jardine P. M. (2012). Relation between soil order and sorption of dissolved organic carbon in temperate subsoils. Soil Sci. Soc. Am. J. 76, 1027–1037. doi: 10.2136/sssaj2011.0340

[ref26] Millard P. Singh B. K. (2010). Does grassland vegetation drive soil microbial diversity? Nutr. Cycl. Agroecosyst. 88, 147–158. doi: 10.1007/s10705-009-9314-3, PMID: 35879955

[ref27] Mtambanengwe F. Mapfumo P. Kirchmann H. (2004). “Decomposition of organic matter in soil as influenced by texture and pore size distribution” in Managing nutrient cycles to sustain soil fertility in sub-Saharan Africa. ed. Bationo A. (Nairobi: Academy Science Publishers (ASP)). 261–276.

[ref011] Mondav R. Woodcroft B. J. Kim E. H. McCalley C. K. Hodgkins S. B. Crill P. M.. (2014). Discovery of a novel methanogen prevalent in thawing permafrost. Nature Communications 5:3212. doi: 10.1038/ncomms4212 24526077

[ref012] Monteux S. Weedon J. T. Blume-Werry G. Gavazov K. Jassey V. E. J. Johansson M.. (2018). Long-term in situ permafrost thaw effects on bacterial communities and potential aerobic respiration. Isme Journal 12, 2129–2141. doi: 10.1038/s41396-018-0176-z 29875436 PMC6092332

[ref28] Ni Z. X. Lu X. F. Huang G. W. (2021). Impact of meteorological factors on Thermokarst Lake changes in the Beilu River basin, Qinghai-Tibet plateau, China (2000–2016). Water. 13:1605. doi: 10.3390/w13111605

[ref013] Nichols J. D. (1984). Relation of organic-carbon to soil properties and climate in the southern great plains. Soil Sci. Soc. Am. J. 48, 1382–1384. doi: 10.2136/sssaj1984.03615995004800060037x

[ref29] Niu F. Cheng G. Ni W. Jin D. (2005). Engineering-related slope failure in permafrost regions of the Qinghai-Tibet plateau. Cold Regions Sci. Technol. 42, 215–225. doi: 10.1016/j.coldregions.2005.02.002

[ref30] Otten W. Hall D. Harris K. Ritz K. Young I. M. Gilligan C. A. (2001). Soil physics, fungal epidemiology and the spread of Rhizoctonia solani. New Phytol. 151, 459–468. doi: 10.1046/j.0028-646x.2001.00190.x

[ref014] Peddle D. R. Franklin S. E. (1993). Classification of permafrost active layer depth from remotely sensed and topographic evidence. Remote Sens. Environ. 44, 67–80. doi: 10.1016/0034-4257(93)90103-5

[ref31] Qin S. Chen L. Fang K. Zhang Q. Wang J. Liu F.. (2019). Temperature sensitivity of SOM decomposition governed by aggregate protection and microbial communities. Sci. Adv. 5:eaau1218. doi: 10.1126/sciadv.aau1218 31309137 PMC6620099

[ref32] Qin S. Q. Kou D. Mao C. Chen Y. L. Chen L. Y. Yang Y. H. (2021). Temperature sensitivity of permafrost carbon release mediated by mineral and microbial properties. Sci. Adv. 7:eabe3596. doi: 10.1126/sciadv.abe3596 34362729 PMC8346221

[ref33] Roberts J. A. (2004). Inhibition and enhancement of microbial surface colonization: the role of silicate composition. Chem. Geol. 212, 313–327. doi: 10.1016/j.chemgeo.2004.08.021

[ref015] Schadel C. Bader M. K. F. Schuur E. A. G. Biasi C. Bracho R. Capek P.. (2016). Potential carbon emissions dominated by carbon dioxide from thawed permafrost soils. Nature Climate Change 6, 950–953. doi: 10.1038/nclimate3054

[ref016] Schuur E. A. G. Bockheim J. Canadell J. G. Euskirchen E. Field C. B. Goryachkin S. V.. (2008). Vulnerability of permafrost carbon to climate change: Implications for the global carbon cycle. Bioscience 58, 701–714. doi: 10.1641/b580807

[ref017] Schuur E. A. G. McGuire A. D. Schadel C. Grosse G. Harden J. W. Hayes D. J.. (2015). Climate change and the permafrost carbon feedback. Nature 520, 171–179. doi: 10.1038/nature14338 25855454

[ref34] Shahzad T. Rashid M. I. Maire V. Barot S. Perveen N. Alvarez G.. (2018). Root penetration in deep soil layers stimulates mineralization of millennia-old organic carbon. Soil Biol. Biochem. 124, 150–160. doi: 10.1016/j.soilbio.2018.06.010

[ref35] Siciliano S. D. Palmer A. S. Winsley T. Lamb E. Bissett A. Brown M. V.. (2014). Soil fertility is associated with fungal and bacterial richness, whereas pH is associated with community composition in polar soil microbial communities. Soil Biol. Biochem. 78, 10–20. doi: 10.1016/j.soilbio.2014.07.005

[ref36] Six J. Callewaert P. Lenders S. De Gryze S. Morris S. J. Gregorich E. G.. (2002). Measuring and understanding carbon storage in afforested soils by physical fractionation. Soil Sci. Soc. Am. J. 66, 1981–1987. doi: 10.2136/sssaj2002.1981

[ref37] Sun N. L. Zhong J. H. Hao B. Ge Y. Z. Swennen R. (2020). Sedimentological and diagenetic control on the reservoir quality of deep-lacustrine sedimentary gravity flow sand reservoirs of the upper Triassic Yanchang formation in southern Ordos Basin, China. Mar. Pet. Geol. 112:29. doi: 10.1016/j.marpetgeo.2019.104050

[ref38] Treat C. C. Wollheim W. M. Varner R. K. Grandy A. S. Talbot J. Frolking S. (2014). Temperature and peat type control CO2 and CH4 production in Alaskan permafrost peats. Glob. Chang. Biol. 20, 2674–2686. doi: 10.1111/gcb.12572, PMID: 24616169

[ref39] Vieira S. Sikorski J. Dietz S. Herz K. Schrumpf M. Bruelheide H.. (2020). Drivers of the composition of active rhizosphere bacterial communities in temperate grasslands. ISME J. 14, 463–475. doi: 10.1038/s41396-019-0543-4, PMID: 31659233 PMC6976627

[ref40] Wang G. X. Li Y. S. Wu Q. B. Wang Y. B. (2006). Impacts of permafrost changes on alpine ecosystem in Qinghai-Tibet plateau. Sci. China. Ser. D Earth Sci. 49, 1156–1169. doi: 10.1007/s11430-006-1156-0

[ref41] Wu Q. B. Hou Y. D. Yun H. B. Liu Y. Z. (2015). Changes in active-layer thickness and near-surface permafrost between 2002 and 2012 in alpine ecosystems, Qinghai-Xizang (Tibet) plateau, China. Glob. Planet. Change. 124, 149–155. doi: 10.1016/j.gloplacha.2014.09.002

[ref42] Xia Q. Rufty T. Shi W. (2020). Soil microbial diversity and composition: links to soil texture and associated properties. Soil Biol. Biochem. 149:13. doi: 10.1016/j.soilbio.2020.107953

[ref43] Yu Q. H. Fan K. You Y. H. Guo L. Yuan C. (2015). Comparative analysis of temperature variation characteristics of permafrost roadbeds with different widths. Cold Reg. Sci. Technol. 117, 12–18. doi: 10.1016/j.coldregions.2015.05.002

[ref018] Zhang T. Barry R. G. Knowles K. Heginbottom J. A. Brown J. (1999). Statistics and characteristics of permafrost and ground-ice distribution in the Northern Hemisphere. Polar Geography 23, 132–154. doi: 10.1080/10889379909377670

[ref019] Zhang Y. G. Liu X. Cong J. Lu H. Sheng Y. Y. Wang X. L.. (2017). The microbially mediated soil organic carbon loss under degenerative succession in an alpine meadow. Mol Ecol. 26, 3676–3686. doi: 10.1111/mec.14148 28429833

[ref44] Zhang X. F. Xu S. J. Li C. M. Zhao L. Feng H. Y. Yue G. Y.. (2014). The soil carbon/nitrogen ratio and moisture affect microbial community structures in alkaline permafrost-affected soils with different vegetation types on the Tibetan plateau. Res. Microbiol. 165, 128–139. doi: 10.1016/j.resmic.2014.01.002, PMID: 24463013

[ref45] Zhang X. F. Zhao L. Xu S. J. Liu Y. Z. Liu H. Y. Cheng G. D. (2013). Soil moisture effect on bacterial and fungal community in Beilu River (Tibetan plateau) permafrost soils with different vegetation types. J. Appl. Microbiol. 114, 1054–1065. doi: 10.1111/jam.12106, PMID: 23241008

[ref46] Zinger L. Lejon D. P. H. Baptist F. Bouasria A. Aubert S. Geremia R. A.. (2011). Contrasting diversity patterns of Crenarchaeal, bacterial and fungal soil communities in an alpine landscape. PLoS One 6:7. doi: 10.1371/journal.pone.0019950 PMC3093402 21589876

[ref47] Zou D. F. Zhao L. Sheng Y. Chen J. Hu G. J. Wu T. H.. (2017). A new map of permafrost distribution on the Tibetan plateau. Cryosphere 11, 2527–2542. doi: 10.5194/tc-11-2527-2017

