# Supplementary material for: Soil texture influences soil bacterial biomass in the permafrost-affected alpine desert of the Tibetan plateau
Source: Front Microbiol. 2022 Dec 12;13:1007194. doi: 10.3389/fmicb.2022.1007194 (PMC9791195; doi:10.3389/fmicb.2022.1007194)
Supplement: Supplementary file 1 [file Presentation_1.pdf]

Supplementary Material

1 Supplementary Figures and Tables

1.1 Supplementary Figures

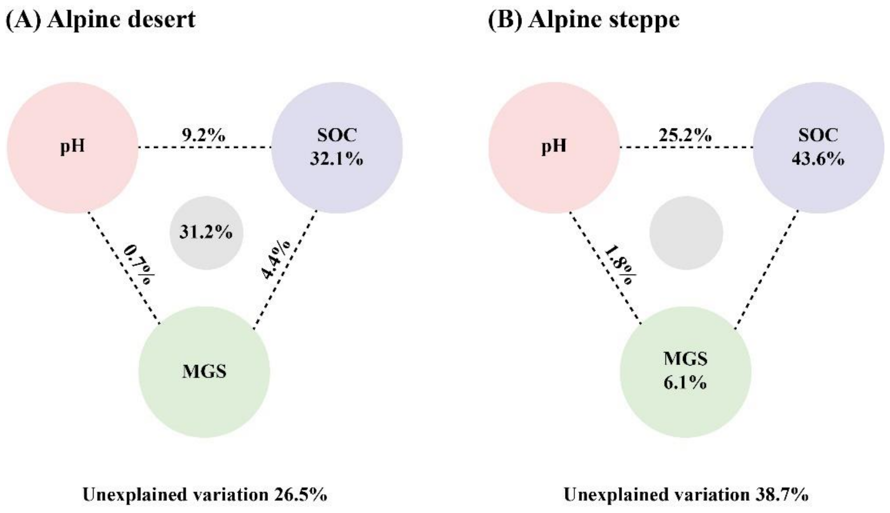

Supplementary Figure 1. Variation partitioning analysis for total PLFAs in the alpine desert (A) and alpine steppe (B).

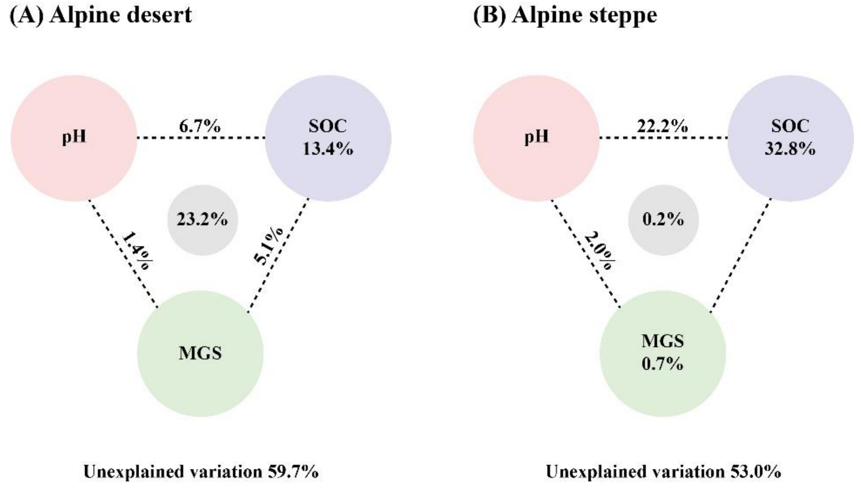

Supplementary Figure 2. Variation partitioning analysis for fungal PLFAs in the alpine desert (A) and alpine steppe (B).

1.2 Supplementary Tables

Supplementary Table 1. Descriptive statistics of physicochemical properties of all the soil samples and different ecosystems.

		Elevation/m	pH	SWC/%	EC (μS/cm)	SOC/‰	Quartz/%	K-Feldspar/%	Plagioclase/%	Calcite/%	Clay mineral/%	MGS (μm)	Coarse sand/%	Fine sand/%	Silt/%	Clay/%
Alpine desert	Min	4679.0	8.3	3.5	39.2	0.4	70.7	0.0	2.4	4.4	0.0	131.1	23.2	44.3	2.3	0.1
	Mean	4709.4	8.7	9.7	61.8	2.1	85.1	1.2	3.9	8.7	1.0	164.1	32.7	59.0	5.4	2.8
	Max	4758.0	9.0	23.4	89.4	8.3	89.2	5.5	5.6	20.4	8.3	187.4	41.0	74.2	15.0	11.8
Alpine steppe	Min	4666.0	8.3	6.7	43.8	1.4	55.5	0.0	3.9	3.5	0.0	75.8	1.5	50.0	2.4	0.2
	Mean	4675.5	8.5	15.2	67.7	4.1	76.1	2.6	7.9	9.3	2.8	123.2	15.6	75.5	8.2	0.8
	Max	4692.0	8.8	29.4	94.1	6.8	88.5	10.5	15.2	17.3	12.6	185.0	40.9	87.7	17.7	1.9

SWC: soil water content, EC: electrical conductivity, SOC: soil organic carbon, TN: total soil nitrogen, MGS: mean grain size.

Supplementary Table 2. Results of mixed effects model showing the effects of depth (0-5cm, 20-30cm and 50-60cm), ecosystems (alpine desert and alpine steppe), and their interactions on soil physicochemical properties, respectively. Asterisks indicate significant influence (** indicate $P < 0.01$; * indicate $P < 0.05$).

	pH	SWC	EC	SOC	Quartz	K-Feldspar	Plagioclase	Calcite	Clay mineral	MGS	Coarse sand	Fine sand	Silt	Clay
Depth	0.553	0.053	0.573	0.841	1.340	2.774	1.026	1.021	0.950	0.270	0.077	0.018	0.739	0.428
Ecosystem	7.352*	4.568*	0.921	5.103*	6.534*	2.984	19.943**	0.080	1.369	18.540**	25.390**	15.800**	2.298	2.565
Depth* Ecosystem	0.268	0.174	0.095	0.493	0.612	0.905	3.702*	0.174	1.080	0.134	0.026	0.045	0.165	0.484

Supplementary Table 3. Pearson correlation coefficients between soil physicochemical properties of different ecosystems.

		Elevation	pH	SWC	EC	SOC	Quartz	K-Feldspar	Plagioclase	Calcite	Clay mineral	MGS	Coarse sand	Fine sand	Silt	Clay		
	Elevation	1	-0.619*	-0.532	0.205	0.673*	0.206	-0.118	0.396	-0.411	-0.243	-0.096	-0.264	0.378	-0.275	-0.306	Elevation	
	pH	0.660**	1	0.204	-0.358	-0.513	0.044	-0.002	-0.205	-0.057	0.124	0.174	0.215	-0.182	-0.038	0.053	pH	
	SWC	-0.722**	-0.580*	1	0.413	0.024	-0.257	-0.341	0.027	0.339	0.126	-0.542	-0.515	0.372	0.213	0.099	SWC	
	EC	-0.635*	-0.427	0.385	1	0.732**	-0.522	-0.191	0.333	0.452	0.430	-0.684*	-0.522	0.184	0.605*	0.510	EC	
	SOC	-0.308	-0.698**	0.214	0.451	1	-0.122	-0.303	0.365	-0.032	0.085	-0.605*	-0.603*	0.431	0.251	0.195	SOC	
	Quartz	0.091	0.066	0.232	-0.256	-0.050	1	-0.588*	-0.528	-0.730**	-0.751**	0.163	-0.068	0.368	-0.624*	-.0588*	Quartz	
	K-Feldspar	0.044	0.169	-0.417	-0.197	-0.178	-0.721**	1	0.305	0.270	0.414	0.587*	0.695*	-0.695*	0.115	0.183	K-Feldspar	
Alpine desert	Plagioclase	-0.061	-0.078	0.456	-0.131	-0.223	0.298	-0.388	1	-0.054	0.187	-0.054	-0.176	0.231	-0.139	-0.167	Plagioclase	Alpine steppe
	Calcite	0.043	0.157	-0.335	0.109	-0.349	-0.875**	0.622*	-0.242	1	0.477	-0.225	0.122	-0.507	0.812**	0.732**	Calcite	
	Clay mineral	-0.198	-0.522*	0.146	0.364	0.932**	-0.098	-0.101	-0.317	-0.333	1	-0.238	-0.007	-0.305	0.630*	0.655*	Clay mineral	
	MGS	0.716**	0.653**	-0.534*	-0.516*	-0.633*	0.056	-0.001	0.056	0.271	-0.618*	1	0.909**	-0.591*	-0.497	-0.393	MGS	
	Coarse sand	0.745**	0.527*	-0.708**	-0.288	-0.280	-0.055	-0.001	-0.370	0.288	-0.248	0.830**	1	-0.872**	-0.096	0.013	Coarse sand	
	Fine sand	-0.313	0.078	0.496	-0.193	-0.402	0.165	0.031	0.678**	-0.138	-0.398	-0.148	-.650**	1	-0.404	-0.495	Fine sand	
	Silt	-0.185	-0.421	-0.070	0.502	0.573*	-0.296	0.089	-0.553*	0.135	0.529*	-.559*	-0.065	-.605*	1	.979**	Silt	
	Clay	-0.251	-0.567*	0.033	0.389	.769**	-0.009	-0.148	-0.426	-0.250	.752**	-0.396	-0.004	-.665**	0.502	1	Clay	

SWC: soil water content, EC: electrical conductivity, SOC: soil organic carbon, TN: total soil nitrogen, MGS: mean grain size. *P < 0.05, ** P < 0.01
